# Antibiotic Prophylaxis for Children with Primary Vesicoureteral Reflux: Where Do We Stand Today?

**DOI:** 10.1155/2008/217805

**Published:** 2008-07-29

**Authors:** Michiel Costers, Rita Van Damme-Lombaerts, Elena Levtchenko, Guy Bogaert

**Affiliations:** ^1^Antibiotic Management Team, University Hospital Leuven Gasthuisberg, Herestraat 49, 3000 Leuven, Belgium; ^2^Department of Pediatric Nephrology and Organ Transplantation, University Hospital Leuven Gasthuisberg, Herestraat 49, 3000 Leuven, Belgium; ^3^Department of Pediatric Nephrology, University Hospital Leuven Gasthuisberg, Herestraat 49, 3000 Leuven, Belgium; ^4^Department of Pediatric Urology, University Hospital Leuven Gasthuisberg, Herestraat 49, 3000 Leuven, Belgium

## Abstract

The main goal of the management of vesicoureteral reflux (VUR) is prevention of recurrent urinary tract infections (UTIs), and thereby prevention of renal parenchymal damage possibly ensuing from these infections. Long-term antibiotic prophylaxis is common practice in the management of children with VUR, as recommended in 1997 in the guidelines of the American Urological Association. We performed a systematic review to ascertain whether antibiotics can be safely discontinued in children with VUR and whether prophylaxis is effective in the prevention of recurrent UTIs and renal damage in these patients. Several uncontrolled studies indicate that antibiotic prophylaxis can be discontinued in a subset of patients, that is, school-aged children with low-grade VUR, normal voiding patterns, kidneys without hydronephrosis or scars, and normal anatomy of the urogenital system. Furthermore, a few recent randomized controlled trials suggest that antibiotic prophylaxis offers no advantage over intermittent antibiotic therapy of UTIs in terms of prevention of recurrent UTIs or new renal damage.

## 1. INTRODUCTION

Vesicoureteral reflux (VUR) is defined as the abnormal, retrograde flow of urine from the urinary bladder into the upper urinary tract. VUR can be primary, caused by an anatomically insufficient vesicoureteric junction, or secondary, due to an infravesical obstruction. VUR affects 1–3% of otherwise healthy children. However, the prevalence rises to 10–20% in children with antenatally detected hydronephrosis, to 30% in siblings of children with known VUR, and to 30–40% in children with a proved urinary tract infection (UTI) [1, 2].

The retrograde flow of urine from the bladder into the ureter may transport bacteria to the upper urinary tract, possibly predisposing these children to febrile UTI, which can result in permanent renal parenchymal damage. Ultimately, renal damage results in reflux nephropathy which could cause hypertension and decreased renal function although the risk seems to be lower than previously thought [3–5].

The clinical presentation of patients with VUR is diverse and dependent on age and gender [6]. Typically, VUR is detected during the evaluation of a child, usually a girl, presenting with UTI [7]. Since the widespread use of prenatal ultrasonography, hydronephrosis is often detected in utero, possibly leading to the diagnosis of VUR in the perinatal period [8]. Neonatal VUR is more common in boys and often associated with congenital renal dysplasia. A history of familial VUR and investigation of an overactive bladder can also lead to the diagnosis of VUR [9, 10].

In 1999, the practice guideline from the American Academy of Pediatrics recommended a renal ultrasound and either a classic radiographic voiding cystourethrography or a direct radionuclide cystography after a first UTI in children aged 2–24 months [11]. However, the recently revised guideline of the National Institute for Health and Clinical Excellence (NICE) constitutes a major departure from this diagnostic strategy [12, 13]. For infants and children without recurrent or atypical UTI, no imaging tests are recommended when they are 6 months or older, and an ultrasound within 6 weeks of the first UTI will suffice when they are younger than 6 months.

Spontaneous resolution of VUR due to the natural elongation of the vesicoureteral junction is possible, especially in patients with the lower grades of VUR or unilateral reflux and in boys; grades I–III reflux resolves at a rate of 13% each year during the first 5 years of follow-up and at 3.5% yearly thereafter, and grade IV reflux resolves at a yearly rate of 5% [14].

The main goal of the management of VUR should be prevention of recurrent febrile UTI, and thereby prevention of the ensuing renal parenchymal damage [6]. The treatment options include intermittent therapy of episodes of UTI, medical therapy with long-term antibiotic prophylaxis, endoscopic therapy, or surgical therapy.

The desire to update our therapeutic algorithm for children with VUR stimulated us to conduct a systematic review of the role of antibiotic prophylaxis in the management of these children. More specifically, we wanted to ascertain whether antibiotics can be safely discontinued and whether prophylaxis is effective in the prevention of recurrent UTIs and renal damage in these patients.

## 2. METHODS

Medline (1966 to June 2008), Embase (1988 to June 2008), and Cochrane Central Register of Controlled Trials were searched using the combined search terms “vesicoureteral reflux” (MeSH) and “antibiotic prophylaxis” (MeSH). National Guideline Clearinghouse, NICE Guidance, and Cochrane Database of Systematic Reviews were searched using the search terms “vesicoureteral reflux” and “urinary tract infection in children.” Reference lists of articles, reviews, and studies were searched for additional studies.

After the search was performed, the titles of all retrieved publications were screened. If the title indicating the paper was potentially relevant, the abstract was reviewed. The full paper was reviewed if the abstract suggested that the paper was indeed relevant. This process was performed by one reviewer (MC Michiel Costers) and thereafter validated by two other reviewers (GB Guy Bogaert and RVDL Rita Van Damme-Lombaerts).

Five uncontrolled studies evaluated the effect of stopping antibiotic prophylaxis in this patient group [15–19].

Five randomized controlled trials (RCTs) and one cohort study that compared antibiotic prophylaxis with no treatment (i.e., surveillance with intermittent therapy of episodes of UTI) for children with VUR were included in this review [20–25].

Two Cochrane systematic reviews and two guidelines on the topic of antibiotic prophylaxis for children with VUR were identified [12, 26–28]. Instead of performing a meta-analysis on this limited number of RCTs, we present the results of these studies.

## 3. RESULTS AND DISCUSSION

Long-term antibiotic prophylaxis remains a common practice in the management of children with VUR. The most commonly used drugs are nitrofurantoin, cotrimoxazole, amoxicillin, and cephalosporins [29, 30]. However, these medications may cause side effects and promote the development of resistant bacteria [22, 23, 25, 31]. Furthermore, the optimal duration of prophylaxis and optimal (low) dose of antibiotic are unclear, and compliance with this long-term treatment is not always assured.

In the 1960's, animal data showed that a UTI in the presence of VUR can cause renal damage. It was then hypothesized that sterilization of the urine could prevent pyelonephritis, and thereby also the resulting parenchymal damage.

At the end of the 1970's, two very small studies indeed suggested that prophylactic antibiotics may prevent recurrent UTIs in children, particularly during the period of prophylaxis. Smellie et al. [32] compared 6–12 months of antibiotic prophylaxis (cotrimoxazole or nitrofurantoin) versus no treatment in 53 children with acute UTI. None of the children in the intervention group had a UTI during the prophylaxis period, while 11 children in the control group presented with a UTI. Twelve months after stopping prophylactic antibiotics, 8 children (32%) in the intervention group compared with 13 (64%) in the control group had suffered from a recurrent UTI.

Lohr et al. [33] performed a crossover study on 18 girls with a history of at least 3 episodes of bacteriuria in the previous year (including 1 girl with VUR). Each child was placed on nitrofurantoin for 6 months and on placebo for a similar period. There were 35 episodes of bacteriuria (4.2 episodes/patient/year) in the patients taking the placebo versus 2 episodes (0.2 episodes/patient/year) in the children taking the antibiotic. Fourteen symptomatic UTIs (1.7 episodes/patient/year) occurred during the placebo periods, and none during the prophylaxis periods.

In 1997, the Pediatric Vesicoureteral Reflux Guidelines Panel of the American Urological Association (AUA) recommended continuous antibiotic prophylaxis as initial therapy for children with reflux grades I–IV [28]. However, this recommendation was based on expert opinion rather than on clear scientific evidence.

### 3.1. Discontinuation of antibiotic prophylaxis

During the following decades, this therapeutic practice has been challenged on multiple occasions. First, several authors demonstrated that in certain circumstances antibiotic prophylaxis can be safely discontinued.

Cooper et al. [15] discontinued antibiotic prophylaxis in 51 children with persistent primary VUR (grades I–IV). All children were old enough to describe the symptoms of UTI (mean age at stop of antibiotics = 8.6 years), and had a minimal or questionable history of true UTI, normal voiding patterns, and kidneys with no significant hydronephrosis or scars. A retrospective chart review revealed 6 episodes (11.8%) of UTI after cessation of prophylaxis (mean follow-up off antibiotics = 3.7 years): 1 case of cystitis and 5 cases of clinically presumptive pyelonephritis. None of the children showed new renal scars on renal ultrasound. However, it should be noted that renal ultrasound has low sensitivity for detection of renal scars.

The retrospective chart review by Thompson et al. [16] of 196 children (mean age at stop of antibiotics = 6 years) who had been withdrawn from prophylactic antibiotics (mean follow-up off antibiotics = 3.4 years) despite persistent reflux (all grades) showed a similar rate of UTIs per patient/year on or off antibiotics (0.29 on versus 0.24 off). Paradoxically, for the 39 children with high-grade reflux IV or V, there was a difference in the rate of UTIs per patient/year seemingly in favor of discontinuation of antibiotic prophylaxis (0.39 on versus 0.18 off). In addition, the rate of new renal scarring on DMSA scan after stop of antibiotics was comparable with the rate during prophylaxis (2.6% on versus 3.6% off).

Hellerstein and Nickell [17] followed (mean follow-up of 3.7 years) 66 children (mean age at stop of antibiotics = 4.4 years for the girls and 3.1 years for the boys) considered at risk for UTI (including 48 children with VUR) after completion of the initial course of prophylactic antibiotics. During the initial course of prophylactic antibiotics, 16 children presented with UTIs, with voiding dysfunction and abnormal kidney(s) being identified as risk factors for these infections. Twenty-eight children had UTI during the follow-up period, but 13 of these children were receiving an antibiotic at the time of the infection. Voiding dysfunction was again identified as a risk factor for infection in this time period.

Al-Sayyad et al. [18] also performed a retrospective chart review of 78 children of 4 years or older with persistent VUR (mean age at stop of antibiotics = 5.7 years) and with reflux grade less than IV and normal voiding pattern or mild voiding dysfunction, who were taken off antibiotic prophylaxis (mean follow-up off antibiotics = 37.7 months). UTI developed in 9 children (11.5%): 8 cases of cystitis and 1 case of clinically presumptive pyelonephritis. None of the children had new renal scarring detected on renal ultrasound.

Fifty-four children (mean age at stop of antibiotics = 6 years) with persistent VUR (all grades, but only 2 patients had high-grade reflux IV or V at the stop of antibiotics) were followed prospectively after discontinuation of antibiotic prophylaxis (mean follow-up off antibiotics = 4.4 years) in the study by Georgaki-Angelaki et al. [19]. All these children were old enough to describe symptoms of UTI, and had normal voiding patterns, kidneys without hydronephrosis or new scar lesions, and a period of at least 2 years without UTI. The number of symptomatic UTI episodes was similar during the on- and off-prophylaxis periods: 9 (cystitis 3 and pyelonephritis 6) and 8 episodes (cystitis 1 and pyelonephritis 7), respectively. No new scars were detected by DMSA scan at the end of the prophylaxis period (50 children tested) and at the end of the follow-up period (33 children tested). In none of the children, renal function deteriorated.

### 3.2. Antibiotic prophylaxis versus intermittent therapy of episodes of urinary tract infection

The small studies by Reddy et al. [20] and Craig et al. [21] were the first to compare antibiotic prophylaxis with no treatment. In the study by Reddy et al. [20], 43 children with VUR were randomly assigned to one of three groups: daily urine nitrate tests without antibiotic prophylaxis (surveillance), daily urine nitrate tests with antibiotic prophylaxis 3 times a week (intermittent prophylaxis), or daily antibiotic prophylaxis (continuous prophylaxis). The incidence of UTI in the 3 groups was as follows: 1/13, 2/14, and 5/16 in the continuous prophylaxis, intermittent prophylaxis, and surveillance groups, respectively.

In the study by Craig et al. [21], 41 children under 3 months of age with asymptomatic VUR received antibiotic prophylaxis (cotrimoxazole for 3 years) or placebo. Two children in the placebo group (*n* = 20) and no child in the antibiotic group (*n* = 21) developed UTI, and none of the children developed new renal damage on DMSA scan.

The multicenter study of Garin et al. [22] evaluated the role of VUR in causing UTI and renal parenchymal damage in 218 patients after an episode of acute pyelonephritis, and determined whether antibiotic prophylaxis (nitrofurantoin or cotrimoxazole for 1 year) could prevent UTI and renal parenchymal damage in the subgroup of patients with mild or moderate VUR (grades I–III). After 1 year of follow-up, the presence of VUR did not significantly increase the incidence of UTI or renal scarring on DMSA scan. Among the 113 patients with VUR, antibiotic prophylaxis did not result in a clinical advantage to prevent UTI (23.6% on versus 22.4% off prophylaxis) or renal scars (9% on versus 3.4% off prophylaxis). Ironically, recurrent acute pyelonephritis was more frequent in the intervention group than in the control group (12.9% on versus 1.7% off prophylaxis) and in all 7 cases, while on antibiotics the offending bacteria showed resistance to the used antibiotic.

In the updated meta-analysis for the Cochrane Database of Systematic Reviews [26], the authors stated that the studies by Reddy et al. [20] and Garin et al. [22] were unable to demonstrate a difference in the risk for UTI or renal parenchymal damage between intervention and control groups, and also that differences cannot be excluded because of the small number of patients studied so far. Furthermore, they concluded that combined therapy (antibiotic prophylaxis plus surgery) offers no advantages over antibiotic prophylaxis alone in terms of risk for UTI or renal parenchymal damage.

Conway et al. [23] studied a cohort of 611 children aged 6 years or younger with a first episode of UTI and without significant comorbidity to identify risk factors for recurrent UTI and examine the effect of antibiotic prophylaxis on recurrent UTI. Age of 3–5 years and high grade of reflux (IV or V) were identified as risk factors for recurrent UTI; the impact of voiding pattern was not evaluated in this study. They found that antibiotic prophylaxis had no significant effect on the risk of recurrence of UTI (hazard ratio of 1.01), even when stratified by type of antibiotic and stratified for covariates such as sex, race, age, and result of VCUG. Among the 83 children with recurrent UTI, a nested case-control study was performed to determine risk factors for isolation of resistant bacteria. Antibiotic prophylaxis clearly increased the likelihood of the infection being caused by a resistant pathogen (odds ratio of 7.50).

A French multicenter study by Roussey-Kesler et al. [24] evaluated whether antibiotic prophylaxis (cotrimoxazole for 18 months) could reduce the recurrence of UTI in 225 young children with grade I, II, or III VUR. Eighteen months later, recurrence of all UTIs and febrile UTIs was not significantly different between the intervention and control groups: 17% versus 26% (*P* = .15) and 13% versus 16% (*P* = .52), respectively. When patients with grades I–III reflux were analyzed separately, again no significant differences were observed (*P* = .22, *P* = .23, and *P* = .57, resp.). However, prophylaxis significantly reduced UTI in boys (*P* = .013) but not in girls (*P* = .8), and then only in those boys with grade III VUR (*P* = .04).

Finally, an Italian multicenter study by Pennesi et al. [25] assessed the effectiveness of antibiotic prophylaxis (cotrimoxazole for 2 years) in preventing pyelonephritis and in avoiding the occurrence of new scars in 100 children with grade II, III, or IV VUR at first episode of pyelonephritis, who were younger than 30 months. After 2 years of follow-up, 18 children (36%) in the intervention group and 15 children (30%) in the control group had at least 1 pyelonephritis recurrence. Thus, the risk for having at least 1 pyelonephritis recurrence was even slightly higher in the intervention group than in the control group (relative risk of 1.2). While all episodes of pyelonephritis in the control group were caused by sensitive strains of *Escherichia coli*, multiresistant bacteria (all resistant to cotrimoxazole among other antibiotics) were responsible for all infections in the intervention group. Furthermore, the presence of renal scars on DMSA scan was the same in children with or without antibiotic prophylaxis (relative risk of 1.2).

According to the recently revised NICE guideline, antibiotic prophylaxis is not routinely recommended in children after first-time UTI, and should only be considered after recurrent UTI [12, 13].

## 4. CONCLUSION

Despite the lack of evidence for its effectiveness, long-term antibiotic prophylaxis has been a common practice in the management of children with VUR for decades. However, several uncontrolled studies (total of 379 children) indicate that antibiotic prophylaxis can safely be discontinued in a subset of patients, that is, school-aged children with low-grade VUR, normal voiding patterns, kidneys without hydronephrosis or scars, and normal anatomy of the urogenital system.

More importantly, several recent RCTs suggest that antibiotic prophylaxis (with cotrimoxazole) offers no advantage over intermittent antibiotic therapy of UTIs in terms of prevention of recurrent UTIs or new renal damage. However, further research is still warranted in view of the limited number of children (total of 522 children) studied in these five RCTs. Furthermore, children with high-grade VUR have generally been excluded from these studies, and these findings cannot therefore be generalized. Finally, one of the RCTs indicates that boys with grade III VUR benefit from antibiotic prophylaxis, and there is a possibility that other subsets of patients, who will benefit from prophylaxis, will be identified in the future.
